# Consensus and controversies in the definition, assessment, treatment and monitoring of BTcP: results of a Delphi study

**DOI:** 10.1007/s12094-016-1490-4

**Published:** 2016-02-08

**Authors:** J. Boceta, A. De la Torre, D. Samper, M. Farto, R. Sánchez-de la Rosa

**Affiliations:** 1 Unidad de Hospitalización Domiciliaria y Cuidados Paliativos, Servicio de Medicina Interna, Hospital Universitario Virgen de la Macarena, Avd. Doctor Fedriani, 3, 41071 Seville, Spain; 2 Servicio de Oncología Radioterápica, Hospital Universitario Puerta del Hierro, Madrid, Spain; 3 S. Anestesiología, Clínica del Dolor, Hospital Germans Trias i Pujol, Barcelona, Spain; 4 Medical Department, TEVA Pharma, Madrid, Spain

**Keywords:** Breakthrough cancer pain, Baseline cancer pain, Management, Consensus, Cancer, Delphi

## Abstract

**Introduction:**

There is no unanimous consensus on the clinical features to define breakthrough cancer pain (BTcP). The current project aimed to investigate the opinion of a panel of experts on cancer pain on how to define, diagnose, assess, treat and monitor BTcP.

**Materials and methods:**

A two-round Spanish multi-centre exploratory Delphi study was conducted with medical experts (*n* = 90) previously selected from Medical Oncology Services, Radiation Oncology, Palliative Care/Home Care Teams, and Pain Units. The study intended to seek experts’ consensus and to define a set of recommendations for the management of BTcP.

**Results:**

It was generally agreed that, definition of BTcP implies that baseline pain should be controlled (84 %), although not necessarily with opioids (only 30 %); there must be exacerbations (98.9 %); the duration of each episode should last <1 h (70 %); the intensity of pain ≥7 out of 10 (67.8 %); and the number of flares per day should not be less than four. All participants supported the use of the Davies algorithm for the diagnosis. The use of a ‘Patient Diary’ was highly recommended. The optimal treatment should have a rapid onset, a short-acting analgesic effect (1–2 h) and transmucosal nasal or oral administration. It was considered very important to develop protocols for the management of cancer pain.

**Conclusions:**

The present Delphi study identified a set of recommendations to define, assess and monitor BTcP.

## Introduction

In 1990, Portenoy and Hagen published an article about a specific pain syndrome that is known as “irruptive pain”, or by the term “breakthrough pain”. Breakthrough pain has been defined as a ‘transient exacerbation of pain that occurs either spontaneously, or in addition to a stable and adequately controlled background pain, generally treated with major opioids’ [1]. Later on, in 2004, same authors excluded the condition of opioid treatment of background pain, and defined it as a “transitory exacerbation of pain experienced by a patient who has relatively stable and adequately controlled baseline cancer pain” [2].

The requirement of background pain to be controlled allows us to distinguish BTcP from end-dose pain flares and those flares that occurred during the drug analgesics titration of the background pain. To emphasize these differences, Davies et al. [3] defined BTcP as a “transitory exacerbation of pain that occurs, either spontaneously or associated with predictable factors or not, even though the baseline pain is relatively stable and well controlled.”

A working group from the European Association of Palliative Care suggested, for linguistic reasons, to replace the term “breakthrough pain” with other terms such as “episodic pain” or “transitory pain” [4]. However, the term “breakthrough pain” is frequently used in clinical practice, and will be the one used throughout this paper.

In 2012, several Spanish medical societies––Sociedad Española de Oncología Médica (SEOM); Sociedad Española de Oncología Radioterápica (SEOR), Sociedad Española de Cuidados Paliativos (SECPAL) and Sociedad Española de Dolor (SED)––adopted a consensus document in which the term “breakthrough pain” refers to a sudden and transient exacerbation of pain of high intensity and short duration (typically less than 20–30 min), which appears over the baseline of a stable persistent pain, when this has been reduced to a tolerable level by the fundamental use of strong opioids [5]. This definition, still in force for many experts [6], again raises the controversy that background cancer pain should be controlled with opioids.

To consider that baseline pain is adequately controlled, some authors assume that the average intensity of pain must be less than four on a verbal numerical rating scale (VNRS) or visual analogue scale (VAS) from 0 to 10, and the maximum number of episodes of BTcP should be three per day [7]. However, a recent study described BTcP in a diverse population of cancer patients that included people who suffered up to 24 episodes a day [8], while still considering these episodes as BTcP, and thus assuming that baseline pain is adequately controlled.

Despite all this knowledge, BTcP is currently under-diagnosed and under-treated. An epidemiological study developed by the International Association for the Study of Pain (IASP) about the features of tumour pain, showed large differences among continental geographical areas in terms of the frequency of diagnosis of BTcP [9]. Even today, there is no validated method for assessment, although a proper evaluation should include a complete analysis of the pain history, frequency and duration of episodes, monitoring the intensity of pain using VNRS or VAS scales, type of pain, triggers, previous medication, and effectiveness of rescue therapy as well as a physical examination of the patient [1].

Around 60–90 % of cancer patients die in pain [10]. Regarding therapeutic options, we know that the characteristics that define BTcP––rapid onset, high intensity and short duration––do not fit the mode of action of traditional opioids. The ideal opioid for the treatment of BTcP should have a rapid onset and a short duration of action [11]. At present, fentanyl, which is available for administration in different forms, is the drug best suited to this profile [11, 12].

Those facts enclosed in the definition of BTcP as well as procedures for its diagnosis, assessment and monitoring may influence the choice of a treatment and consequently patient outcomes. Hence the importance of obtaining a consensus on these issues from a broad group of experts in cancer pain.

The aim of this study was to explore the opinion of an expert panel in cancer pain, regarding the clinical features to define BTcP; to understand the procedures and assessment scales and monitoring of BTcP in clinical practice in Spain; to uncover the discrepancies; and to find areas of consensus to develop recommendations for the management of BTcP.

## Materials and methods

The Delphi method is commonly used to obtain the collective vision of a group of experts on a subject, and is able to extract and clarify the judgement of the group [13]. Its predictive capacity is based on the systematic use of intuitive judgement, pronounced by a group of experts, which identifies the degree of consensus and points of disagreement regarding a specific topic. It involves sending a succession of anonymous questionnaires to a group of previously selected experts, to try to get consensus, but providing anonymity to participants. After finishing the first round, each member receives a summary of responses corresponding to the first questionnaire. Then, each expert has to revise their previous answers, compare them with those of the rest of the panellists, and answer the next questionnaire, trying to seek the widest possible consensus [14, 15].

A total of 90 experienced and trained experts on cancer pain, homogenously distributed across Spain, were selected from different units: medical oncology (*n* = 21), radiation oncology (*n* = 19), pain units (*n* = 26) and palliative care/home care (*n* = 24).

The first questionnaire consisted of 44 questions divided into six blocks, each of them with specific objectives (Table 1). The first round of questions (44 in total) investigated the opinion of experts on the criteria for the definition of BTcP, assessment, screening and diagnostic methods, and how the treatment and monitoring should look like. The mean and standard deviation of the responses were calculated for each question and these data were showed in the second round of the questionnaire; in this way each participant knew the averaged values of the responses to the first round, and they could vary their responses at this point.

**Table 1:** Topics and objectives from the first questionnaire

Subject to evaluate	Objective
Definition of breakthrough of cancer pain	Investigate the level of agreement for each purposed definition
BTcP patients	Estimate the number of patients with breakthrough cancer pain
Importance of BTcP and drawbacks in managing	Ascertain the importance of managing breakthrough cancer pain within the frame of each cancer patient
Detection, diagnose and characterization of breakthrough cancer pain	Get knowledge on the methods to detect, diagnose, and evaluate breakthrough cancer pain in routine clinical praxis
Treatment of breakthrough cancer pain	Get knowledge on the prescription criteria to give the optimal therapy to the right patient and on the monitoring of the expected efficacy
Monitoring breakthrough cancer pain	Study the differences followed by the expert panel members to monitoring cancer patients either with or without breakthrough pain

The second round included only those topics that did not reach consensus (26 questions) and was aimed to clarify the most controversial issues generated during the first round, to inquire any additional issue raised by the results of the first questions, and to agree on some general recommendations.

An online application was developed to administer the questionnaires and to share all information related to the study. Participants provided their responses of both rounds of the survey directly in the online application.

### Data analysis

A descriptive study of the variables was carried out according to their type: for numeric variables, measures of central tendency and dispersion (sample size, mean, median, minimum, maximum, standard deviation, 95 % CI, Q1 and Q3) were applied. For the categorical variables, frequency distribution tables and percentages (*n*, %) were provided.

For the evaluation of some of the answers, a 7-point ordinal rating scale of the Likert type (where 1 = strongly disagree/never/never recommend, and 7 = strongly agree/always/always recommend) was used.

A pairedwise analysis was carried out by means of a Student’s *t* test to analyse the change of the responses between the two rounds. A two-sided 0.05 significance level was set.

## Results

### On the definition, diagnose and management of BTcP

**Fig. 1:**
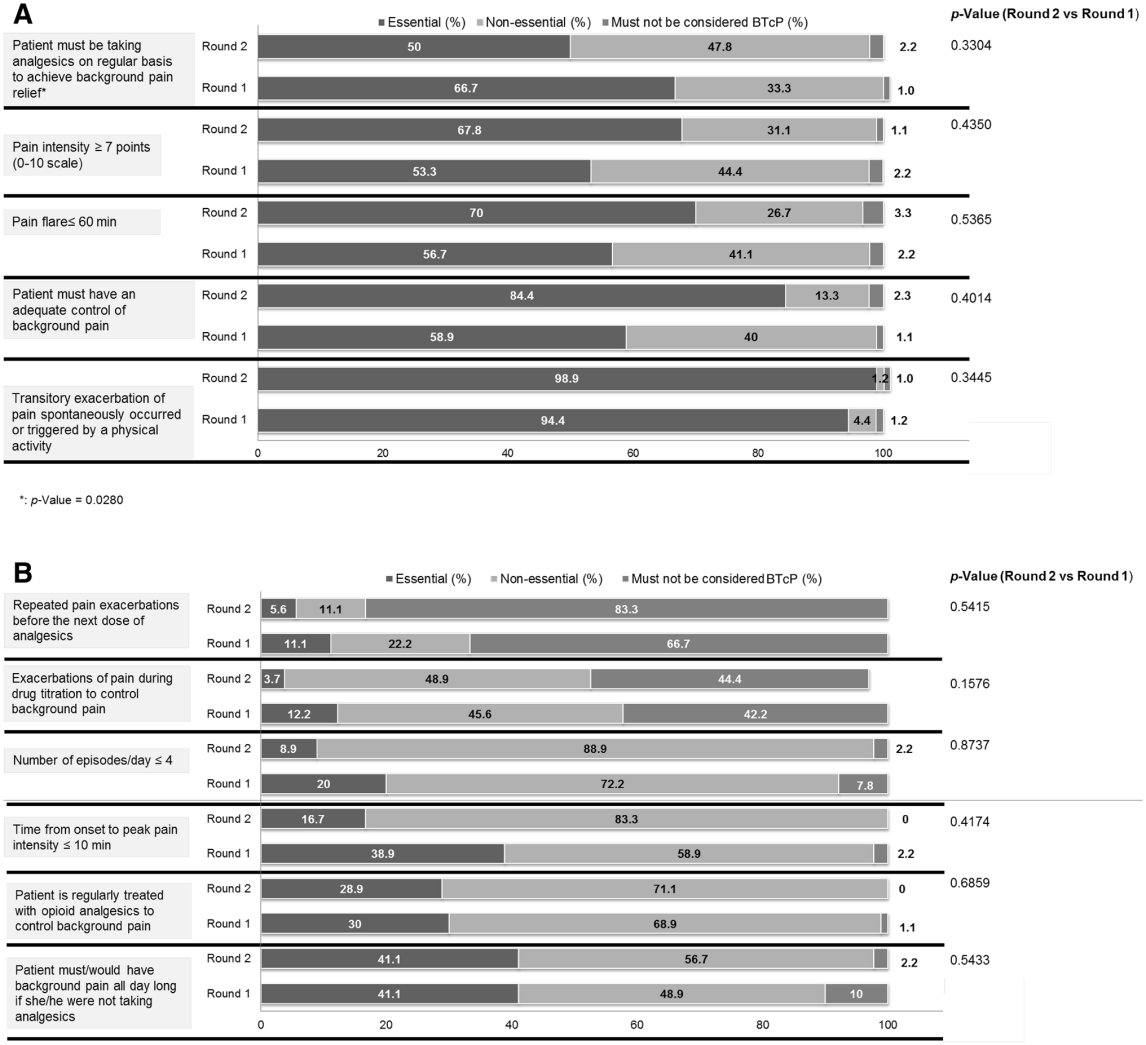
Participants (*n* = 90) responded to the question of the characteristics to be conveniently considered to define BTcP. Each topic was classified as ‘essential’, ‘non-essential’ and ‘must not be considered’. Results obtained from the two rounds of the Delphi study are expressed as a percentage (%)

Half of the panel understood that the patient must be taking analgesics on regular basis to achieve background pain relief, and 84 % stated the importance of an adequate control of cancer pain in the definition of BTcP (Fig. 1a). Likewise, most panellists (98.9 %) defined BTcP as the occurrence of spontaneous or incidental exacerbations of pain. The average duration of each episode must be ≤60 min and pain intensity ≥7 points out of 10. Only 30 % of respondents agreed on the mandatory nature of taking opioid analgesics to define background pain (Fig. 1a, b). Repeated exacerbations of pain before the next dose of analgesics were not classified as BTcP (Fig. 1b).

**Fig. 2:**
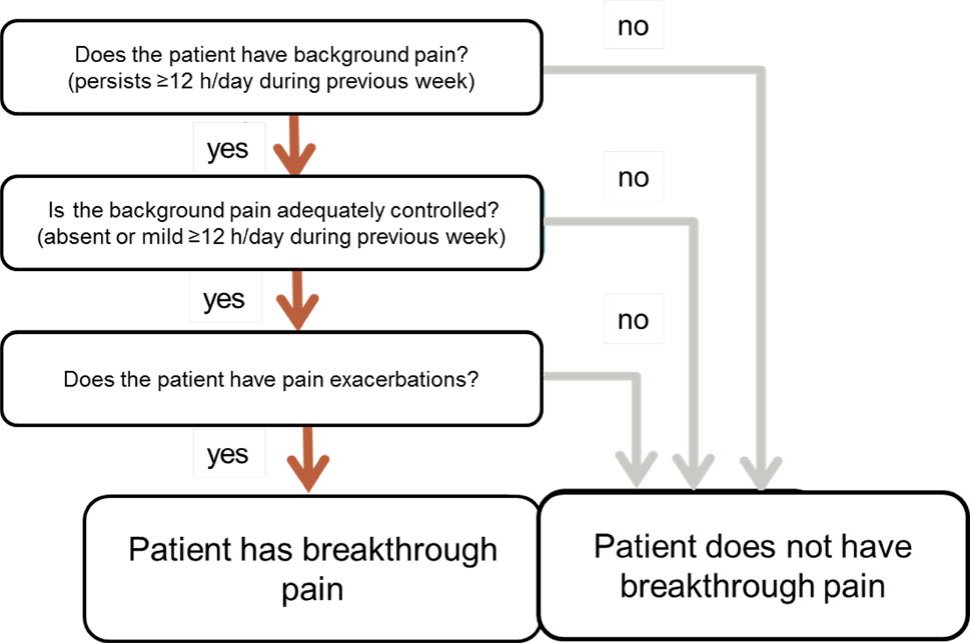
Davies diagnostic algorithm

In the first survey, 34 % of participants recognized not to use the Davies algorithm (Fig. 2) mainly because of the lack of awareness. In the second survey, the question was reformulated and 100 % admitted to ask their patients, and also advise to do that, about the parameters of the Davies algorithm, even if it is not used as an algorithm as such.

Around 68 % of experts confirmed to use a sort of ‘Patient Diary’. Among the parameters to be collected on the diary, the most voted were date and time of each episode, its duration and intensity, the administered rescue dose, time to/degree of pain relief, and side effects, including the recommendation to contact the doctor if more than three rescues per day were required (together with a contact phone number).

To better characterize BTcP, registration of pain analgesics taken by the patient was deemed as the most important factor to be gathered on the clinical history (average Likert score of 6.7 out of 7). Indeed, this item was the most frequently asked to the patient (88.9 %) (Table 2). Other important factors were: the number of flares per day or week, the adherence to pain treatment (both with an average Likert score of 6.6); duration (6.4) and intensity (6.5) of flares; either spontaneous occurrence of flares or triggered by some activity (6.5); and the efficacy of analgesics (6.4). Some important factors to characterize BTcP were hardly taken into account though. Just over 50 % of the experts always ask about the impact of pain on daily life, and only 43.3 % try to discover in a systematic way the strategies that patient uses to relieve pain (Table 2).

**Table 2:** Participants (*n* = 90) respond to the questions about how often they ask to the patient and the importance of several characteristic factors of BTcP (see Likert scores)

*n* = 90 participants	Always (%)	Almost always (%)	Sometimes (%)	Rarely/hardly ever/never	Likert score (round 2)
Pain analgesics taken by the patient*	88.9	10.0	0.6	0.5	6.7
Localization of pain	87.8	10.0	1.2	1.0	6.3
Number of flares per day and/or week	83.3	14.4	1.1	1.2	6.6
Efficacy of drug analgesics*	82.2	12.2	4.4	1.2	6.4**
Intensity of flares (scored with VAS or VNRS)	76.7	16.7	4.4	3.3	6.5
Adherence to the pain therapy	75.6	18.9	3.3	2.2	6.6
Spontaneous or triggered occurrence of pain	75.6	20.0	2.2	2.2	6.5
Irradiation of pain	75.6	15.6	6.7	2.1	6.1
Duration of each flare*	65.6	27.8	5.7	0.9	6.4
Impact of flare on night-sleep	58.9	20.0	16.7	0.4	6.2
Time from start of pain to the highest peak of intensity	57.8	23.3	15.6	3.3	6.0
Similarity (or not) between BTcP and cancer background pain	55.6	32.2	6.7	5.5	5.8

When indicated, data are shown as a percentage and Likert scale ranged from 1 (strongly disagree) to 7 (strongly agree)

*VAS* visual analogue scale, *VNRS* visual numerical rating scale

* *p* value = 0.0430

** Statistical differences among the surveyed medical specialties were observed

**Fig. 3:**
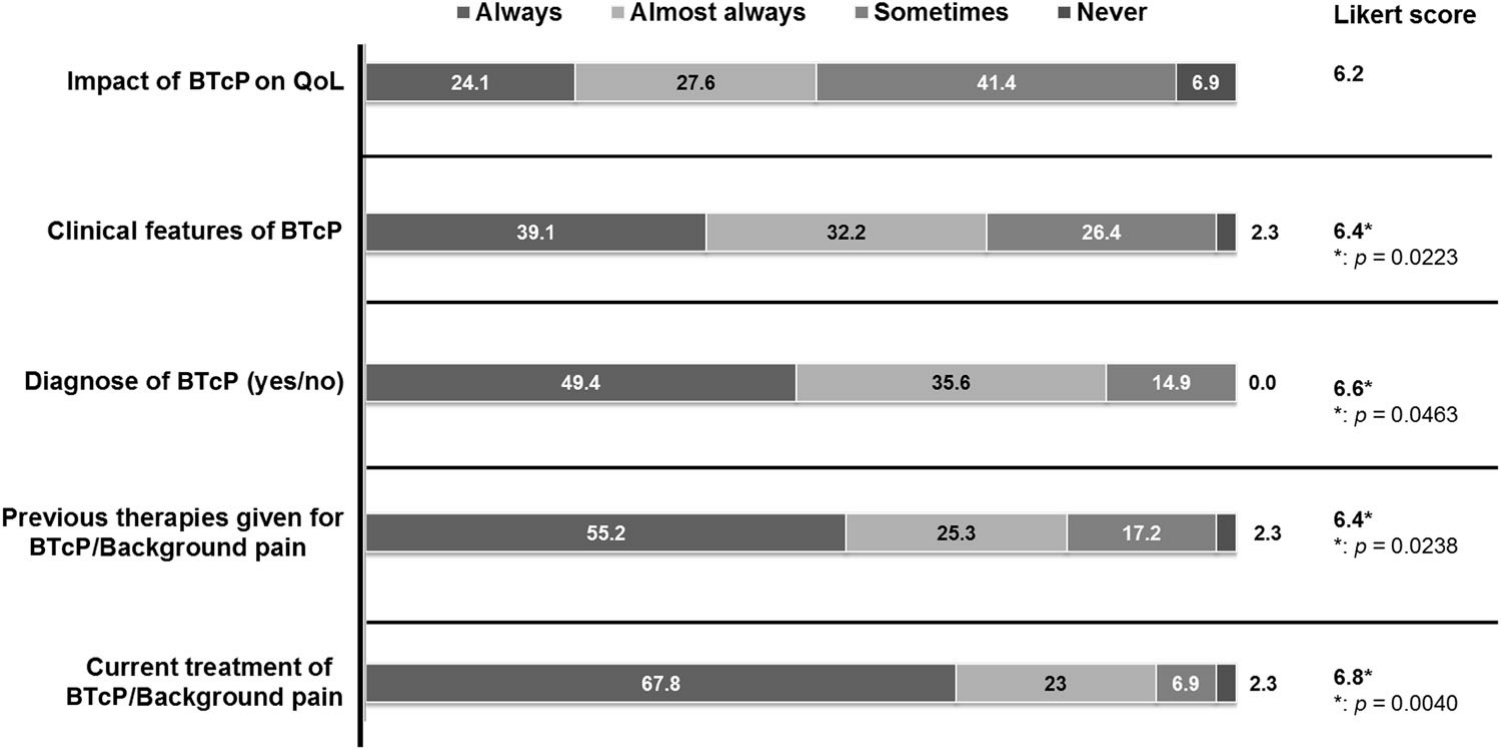
Participants (*n* = 90) respond to the questions about what information (items on the left edge) should be written down on the clinical history of the patient and the importance of each of these items (see the Likert score column). When indicated, data are shown as a percentage. Likert score ranged from 1 (strongly disagree) to 7 (strongly agree). *Statistical differences among the surveyed medical specialties were observed

Participants responded about several topics to be noted down on the clinical history of each patient and categorized their importance (Fig. 3). Most experts agreed on the importance (Likert score of 6.8 out of 7.0) to record the medication for baseline and breakthrough pain. It was also stated the convenience to register the clinical features (71.3 %) and the diagnostic of BTcP (85 %), and to a lesser extent (51.7 %) its impact on quality of life.

### On the treatment and monitoring of BTcP

**Fig. 4:**
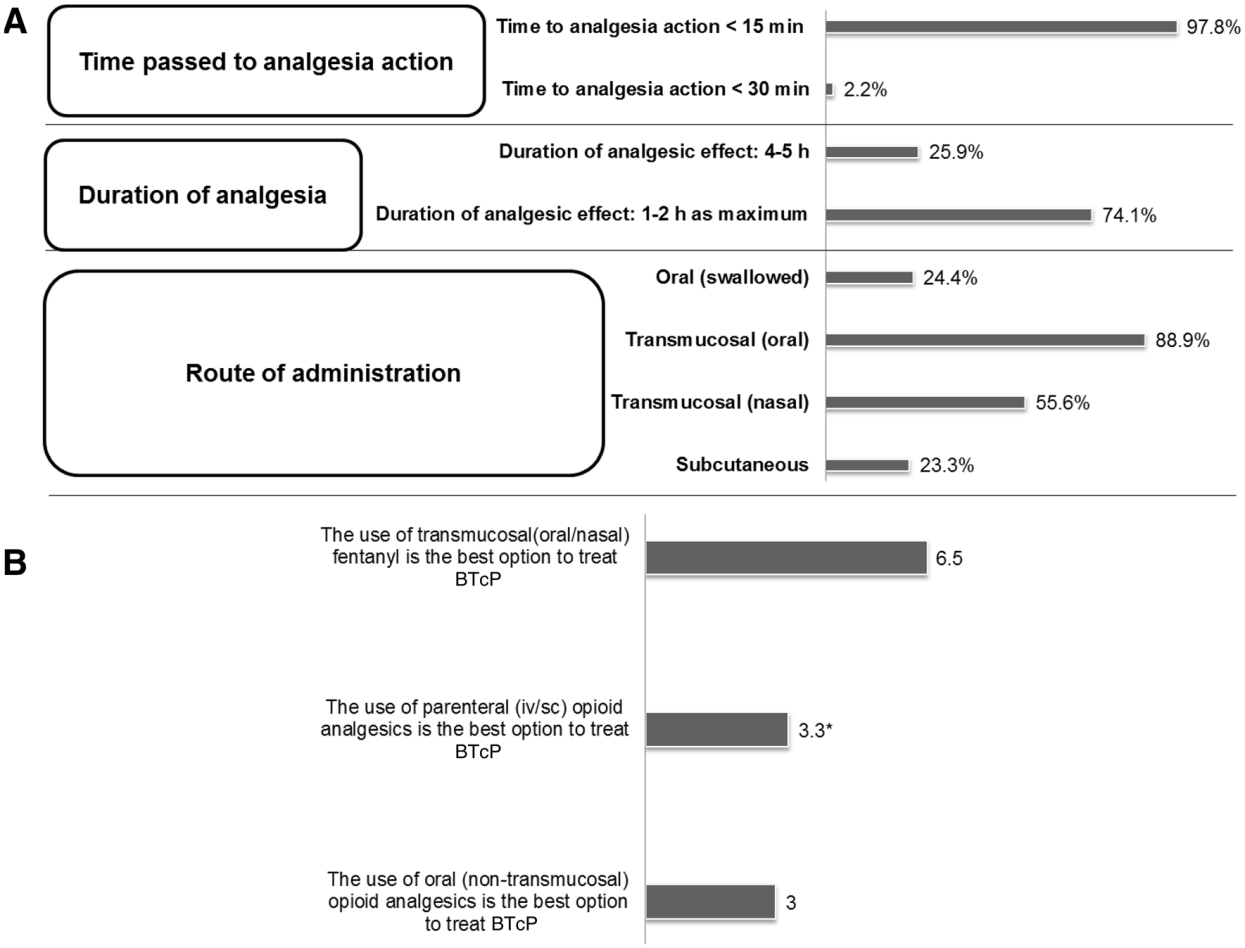
Participants (*n* = 90) respond to the characteristics of the ideal treatment to manage BTcP (**a**) and the most recommended breakthrough analgesic drug (**b**). When indicated, data are shown as a percentage (**a**). The best medication (**b**) was scored using a Likert scale ranged from 1 (strongly disagree) to 7 (strongly agree). *Statistical difference among medical specialties (*p* = 0.0086)

In the first questionnaire, participants were asked about the ideal treatment of BTcP (Fig. 4a). It was mostly reported that the ideal time to onset of analgesic effect should be than 15 min (97.8 %), and duration of the analgesic effect should last maximum of 1–2 h (74.1 %). The panel considered transmucosal fentanyl (oral or nasal forms) as the best option to treat BTcP (Fig. 4b).

**Fig. 5:**
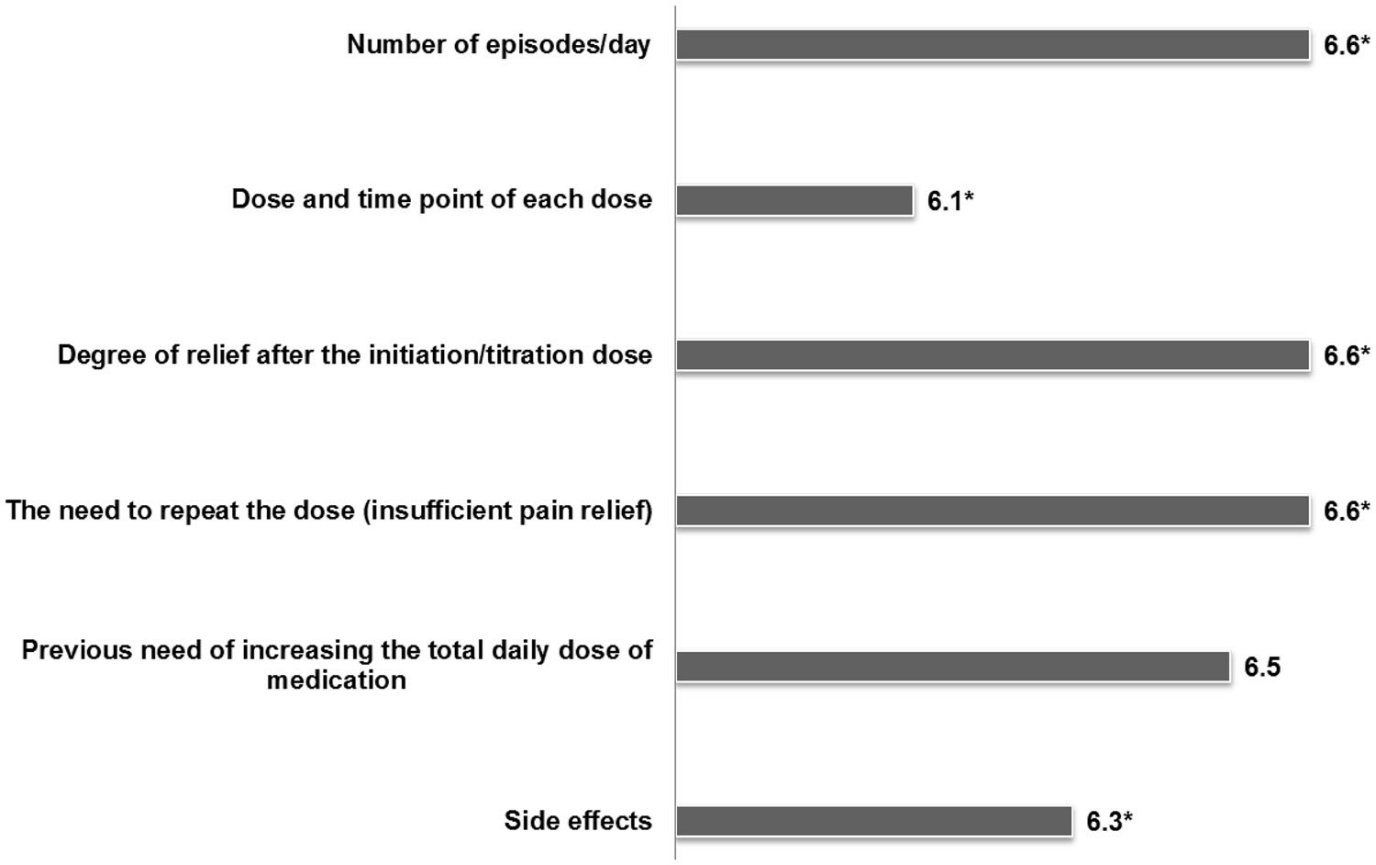
Participants (*n* = 90) were asked to score the importance of several items to be registered for the titration of BTcP medication. Items were scored using a Likert scale ranged from 1 (unimportant) to 7 (extremely important). *Statically significant differences were observed among medical specialties

In the first round, initiation and duration of analgesia, and the route of administration were the most determinant criteria to select a drug for BTcP (Table 3). However, ease of titration, clinical features of each patient and the need of social support to the patient gained more importance in the second survey. After selecting a BTcP drug, many experts (86 %) would carry out a drug titration schedule and almost all (94 %) draft instructions for self-titration at home. The number of flares per day, the need to repeat the dose due to an insufficient relief, and the degree of relief were the highest-scored items to be registered for the titration of BTcP medication (Fig. 5).

**Table 3:** Participants (*n* = 90) were asked to score the importance of several items to be considered for the prescription of the future BTcP medication

Item	Likert score (round 1)	Likert score (round 2) *p* value (round 2 vs round 1)
Initiation of analgesia action	6.5	
Route of administration	6.3	
Duration of analgesia action	6.2	
Ease of titration	6.1	6.4 (*p* = 0.0020)
Clinical features of patients	5.9	6.2 (*p* = 0.0157)
Social support of the patient	5.5	5.9 (*p* = 0.0376)
Pharmacokinetic properties	5.3	
Patient is treated with opioid analgesics to control background pain*	4.9	
Drug availability at the hospital	3.9	

Bars show the results obtained from the first round of the Delphi study. When indicated, those items with significant differences between Round 2 and Round 1 are also shown. Items were scored using a Likert scale ranged from 1 (unimportant) to 7 (extremely important)

* Patients taking at least 60 mg/day oral morphine, 25 μg/h transdermal fentanyl, 30 mg/day oxycodone, 8 mg/day oral hydromorphone or an equivalent dose of other opioid analgesics for a week or longer

**Fig. 6:**
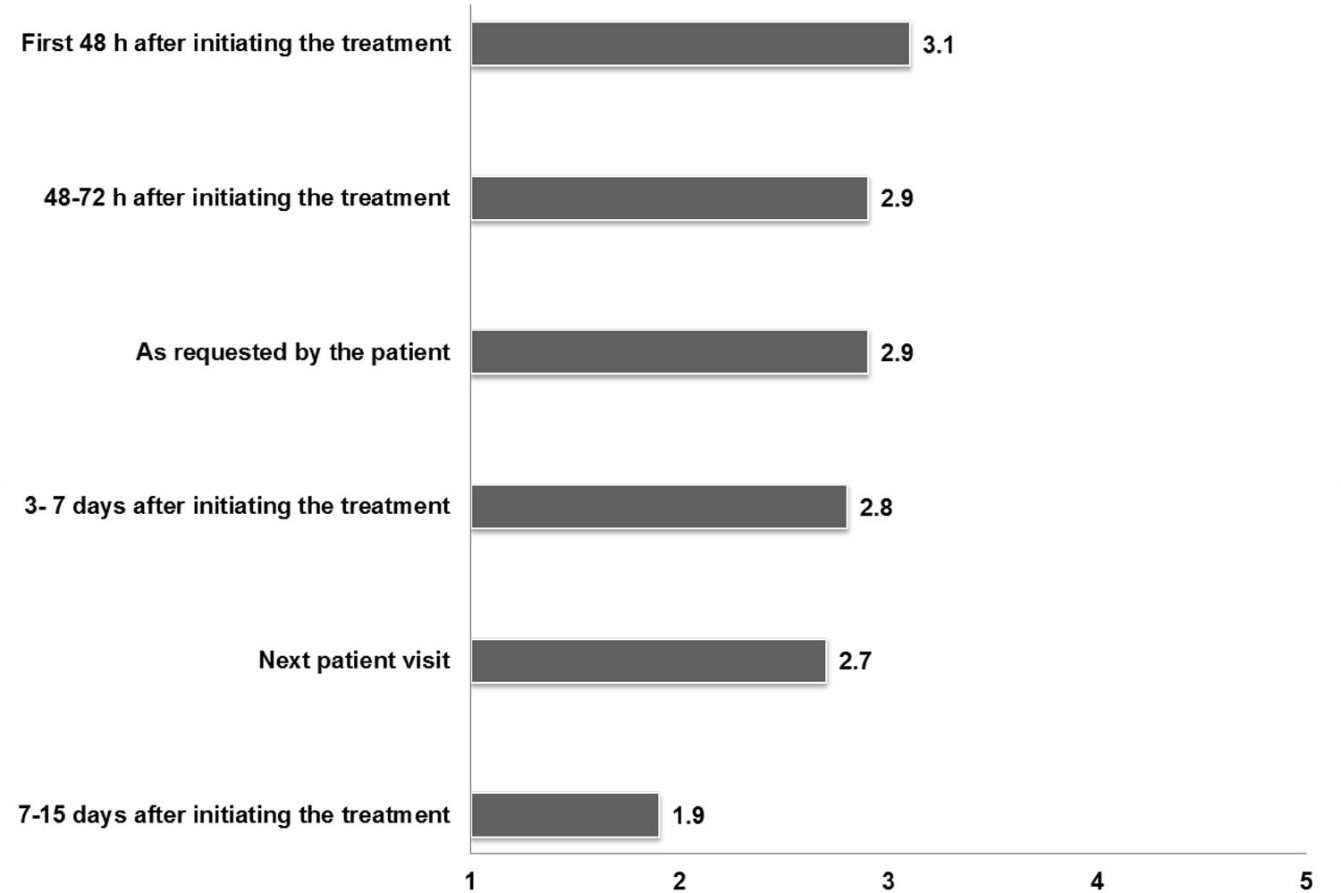
Participants (*n* = 90) responded about the time-frame for the first follow-up visit after initiating the treatment of BTcP. Possible responses were scored using a Likert scale ranged from 1 to 5 (1, in no case– 5, in all cases)

In the first round, and regarding the timeframe for the first follow-up visit, each period was evaluated on a Likert scale of 1–5, where 1 was “in no case” and 5 “in all cases” (Fig. 6). After the second round, experts recommended that the first contact with the patient (including by telephone) should be performed within the first 48 h following the initiation/titration period, and follow-up of patients should be done simultaneously to the scheduled visits and/or whenever requested by the patient.

## Discussion

The Delphi technique chosen for this consensus study has been successfully used in other research studies of palliative care [16] to establish tools for evaluating BTcP, as in the recent Alberta report [17], and it seems to be appropriate for the purposes of this study. The expert panel members were selected to achieve a fair distribution across the four professional profiles involved in the management of BTcP. All Spanish regions were represented in the study. However, the level of participation differed among them, with Catalonia being the least represented.

There is no universally accepted definition of BTcP [18, 19]. Similar to the definitions given by other authors [1–3, 20], the experts consulted agreed to define BTcP as a transitory exacerbation of pain (lasting less than 60 min), which occurs spontaneously or in association with a specific predictable or unpredictable trigger at some time point during the day in cancer patients, despite relatively stable and adequately controlled background pain. There is no agreement on whether the theoretical definition of BTcP might include that background pain is treated with opioids or other analgesics, which is in consistency with the controversy found in the literature for different definitions of BTcP. Only 30 % of the panel judge the use of opioid analgesics to control the background pain to be essential, whereas 50 % stated as mandatory the fact of taking analgesics. The issue of treating breakthrough pain with rapid-onset opioid drugs must be set apart as a simple advisable therapy.

Because end-of-dose pain is the result of an inadequate dose of analgesic or a dosing interval that is too long, the analgesic regimen used to treat baseline persistent pain should be reassessed and modified as necessary. This might explain why end-of-dose pain was not considered BTcP in this study.

The history of the patient with an exacerbation of cancer pain should include a diagnostic method of BTcP. Literature recommends the use of Davies algorithm (Fig. 2) [8], modified from the original published by Davies in 2009 [3]. Although 66 % of respondents recognise not to use it properly as an algorithm, almost all panellists ask, and recommend asking, about the three issues enclosed in the algorithm: the occurrence of background pain, its adequate control, and pain exacerbations.

To enable proper monitoring of BTcP, participants in the study recommend the use of a ‘Patient Diary’. Information to be mainly recorded in such a diary was the date and time of each episode, its duration and intensity, the administered rescue dose, time to relief and degree of relief, side effects, and information about the drug titration phase.

Many authors suggest that BTcP should be assessed before the start of a certain therapy and afterwards, at regular intervals until an adequate pain control is achieved [2]. Most panellists recommended that patients should contact their doctor 48 h after initiated the therapy, and later if they need more than three rescue doses per day.

In our study, the greatest importance is given to the knowledge of the opioid analgesics taken by the patient for pain, separately from those that are prescribed to control BTcP. The number of flares per day or week, their duration and intensity, the degree of adherence to treatment and its efficacy, and the type of breakthrough pain (spontaneous or incidental) were other factors stated by the experts. This was aligned to that reported by literature. Previous studies report that a proper assessment of breakthrough pain should include frequency and duration of episodes, intensity and type of pain, precipitating factors, previous medication and effectiveness of rescue therapy [21].

Surprisingly, there is no unanimity about the impact of BTcP on the quality of life of patients. Some studies show the great impact that BTcP has on mood and functional status [22]. BTcP is described as a frequent complication, of sudden onset, short duration and moderate to severe intensity, and with a very negative impact on quality of life at physical (disability, insomnia), psychological (anxiety, depression) and social levels (unemployment, social isolation) [23]. This contradiction might be explained by the lack of time of physicians, or by the absence of specific protocols to record all these data.

Breakthrough pain is not a single condition, but an assortment of very different conditions. Professionals identified the importance to develop protocols for the management of cancer pain, including BTcP. Primary care and nursing should be also involved in the preparation of such protocols.

Regarding the characteristics of the treatment, participants believe that the ideal therapy of BTcP must meet these conditions: a short-acting effect (the time to onset of analgesic effect is less than 15 min), the analgesia lasts as longest as 1–2 h, and the route of administration is transmucosal (oral or nasal). In the present study, immediate-release fentanyl is chosen by the majority of specialists as the drug of choice to treat BTcP. This is due to its pharmacokinetic properties as well as its rapid onset of action. The selection of one or another presentation and route of administration (oral or nasal) will depend primarily on the clinical situation and the personal preferences of the patient. In the literature, the treatment of BTcP involves strategies such as the treatment of cancer disease, modification of the baseline analgesic treatment, non-pharmacological interventions, and an appropriate rescue medication [24]. The ideal opioid for this treatment should have a rapid onset of action (short time interval between administration and the presence of minimum effective concentrations in the bloodstream), be sufficiently powerful, and have a short duration of action [25].

According to the presentation “New approaches to the diagnosis and treatment of breakthrough cancer pain consensus document” at the XIV Simposio de Revisiones en Cáncer (Madrid), the rescue dose is not related to the baseline opioid dosage. Our panel believes it is essential to carry out titration of the rescue medication, which can be done at patient’s home by means of a previously drafted set of instructions, and recording the recommended information in the ‘Patient Diary’ in a particular manner during this phase: number of flares per day, any need to repeat the dose because of insufficient pain relief, and the degree of relief obtained. In this way the efficacy and tolerability of the treatment can be evaluated and any changes in the nature of BTcP can be recognised [6]. We recommend a first assessment contact within the first 48 h of the drug titration schedule. Further monitoring of BTcP can be managed using routine scheduled visits to the patient and/or subject to patient’s request.

## Conclusions

Regarding the characteristics that best define BTcP, there was a broad agreement that the baseline pain should be controlled, but not necessarily with opioids; there must be exacerbations; the duration of the episode should be less than an hour, and the intensity of pain greater than 7 out of 10.

There was no agreement about whether the number of daily episodes of BTcP has to be less than four to consider that the baseline pain is controlled. The need to treat background pain with opioids was also a matter for controversy.

There is consensus in considering that BTcP is not the same as end-of-dose effect.

Questions encompassed in the Davies algorithm should be asked to diagnose BTcP.

The best tool for assessing and monitoring BTcP is the ‘Patient Diary’, either as a standardized document, or in the form of some generic recommendations on patient outcome records for the referral to the doctor. This should record the date and time of each episode, duration and intensity of each flare, administered rescue dose, time to pain relief, degree of relief and side effects. ‘Patient Diary’ should also include instructions for titration, the recommendation to contact the doctor within the first 48 h of initiating the therapy and/or if more than three rescues per day are required, and a contact phone number. The diagnose of BTcP should be logged in the patient’s clinical record. Protocols for the diagnosis, assessment and monitoring of BTcP are needed and these should take into account the roles of all the professionals involved, including primary care and nursing teams.

The optimal drug to treat BTcP should have a rapid onset of action (15 min or less), short-acting effect (≤2 h) and an easy route of administration (transmucosally). Fentanyl, either in its oral or nasal transmucosal form is well established as the best active substrate that fits better this profile.

Drug titration can be done at patient’s home. Instructions are advised to be provided, so that the patient gathers the information from the titration phase to be included on the patient diary: number of flares per day, need to repeat the dose because of an insufficient relief from the flare, and degree of relief.

## References

[1] Caraceni A, Martini C, Zecca E, Portenoy RK, Working Group of an IASP Task Force on Cancer Pain (2004). Breakthrough pain characteristics and syndromes in patients with cancer pain––an international survey. Palliat Med.

[2] Portenoy RK, Forbes K, Lussier D, Hanks G, Doyle D, Hanks G, Cherny N, Calman K (2004). Difficult pain problems: an integrated approach. Oxford textbook of palliative medicine.

[3] Davies AN, Dickman A, Reid C, Stevens AM, Zeppetella G (2009). The management of cancer-related breakthrough pain: recommendations of a task group of the Science Committee of the Association for Palliative Medicine of Great Britain and Ireland. Eur J Pain.

[4] Mercadante S, Radbruch L, Caraceni A, Cherny N, Kaasa S, Nauck F, Steering Committee of the European Association for Palliative Care (EAPC) Research Network (2002). Episodic (breakthrough) pain: consensus conference on an expert working group of the European Association for Palliative Care. Cancer.

[5] Consenso sobre dolor irruptivo oncológico (2012). http://www.actasanitaria.com/fileset/doc_65005_FICHERO_NOTICIA_49088.pdf. Accessed 20 Jan 2016.

[6] Escobar Y, Biete A, Camba M, Gálvez R, Mañas A, Rodíguez-Sánchez CA (2013). Diagnóstico y tratamiento del dolor irruptivo oncológico: recomendaciones de consenso. Rev Soc Esp Dolor.

[7] Grupo de trabajo de la Guía de Práctica clínica sobre cuidados paliativos. Dosificación de opioides en el Dolor. En: Guía de práctica clínica sobre cuidados paliativos 2008. http://www.euskadi.net/contenidos/informacion/osteba_publicaciones/es_osteba/adjuntos/GPC_Paliativos_resum.pdf. Accessed 20 Jan 2016.

[8] Davies A, Buchanan A, Zeppetella G, Porta-Sales J, Likar R, Weismayr W (2013). Breakthrough cancer pain: an observational study of 1000 European oncology patients. J Pain Symptom Manage.

[9] Ali G, Kopf A, Kopf Andreas, Patel Nilesh B (2010). Breakthrough pain, the pain emergency, and incident pain. Guide to pain management in low-resource settings.

[10] Brennan F, Carr DB, Cousins M (2007). Pain Management: a fundamental human right. Anesth Analg.

[11] Porta-Sales J, Garzón Rodríguez C, Julià Torras J, Casals Merchán M (2010). Dolor irruptivo en cáncer. Med Clin (Barc).

[12] Escobar Álvarez Y, Biete i Solà A, Camba Rodríguez M, Gálvez Mateos R, Mañas Rueda A, Rodríguez Sánchez CA (2014). Diagnóstico y tratamiento del dolor irruptivo oncológico: recomendaciones de consenso. Rev Soc Esp Dolor.

[13] Booberg AL, Morris-Khoo SA (1992). The Delphi method: a review of methodology and an application in the evaluation of a higher education program. Can J Prog Evaluat.

[14] Hasson F, Keeney S, McKenna H (2000). Research guidelines for the Delphi survey technique. J Adv Nurs.

[15] Williams PL, Webb C (1994). The Delphi technique: a methodological discussion. J Adv Nurs.

[16] Jacobsen R, Møldrup C, Christup L (2008). Clinical rationale for administering fentanyl to cancer patients: two Delphi surveys of pain management experts in Denmark. J Opioid Manag.

[17] Hagen NA, Stiles C, Nekolaichuk C, Biondo P, Carlson LE, Fisher K (2008). The Alberta breakthrough pain assessment tool for cancer patients: a validation study using a Delphi process and patient think-aloud interviews. J Pain Symptom Manage.

[18] Davies AN (2011). The management of breakthrough cancer pain. Br J Nurs.

[19] Dickman A (2011). Integrated strategies for the successful management of breakthrough cancer pain. Curr Opin Support Palliat Care.

[20] Manchikanti L, Singh V, Caraway DL, Benyamin RM (2011). Breakthrough pain in chronic non-cancer pain: fact, fiction, or abuse. Pain Physician.

[21] Cánovas L, Rodríguez-Rodríguez AB, Castro M, Pérez-Arviza L, López-Soto C, Román R (2012). Tratamiento del dolor irruptivo. Rev Soc Esp Dolor.

[22] Abernethy A, Wheeler J, Fortner B (2008). A health economic model of breakthrough pain. Am J Manag Care.

[23] Skinner C, Thompson E, Davies A (2006). Clinical features. Cancer related breakthrough pain.

[24] Zeppetella G, Ribeiro MD (2006). Opioids for the management of breakthrough (epi-sodic) pain in cancer patients. Cochrane Database Syst Rev.

[25] Virizuela JA, Escobar Y, Cassinello J, Borrega P (2012). Treatment of cancer pain: spanish Society of Medical Oncology (SEOM) recommendations for clinical practice. Clin Transl Oncol.

